# Vermont State Dental Society

**Published:** 1893-09

**Authors:** 


### Vermont State Dental Society.

The seventeenth annual meeting of the Vermont State Dental Society, held at the Welden House, St. Alban's, March 15, 16 and 17, was very interesting and well attended, there being fully one hundred dentists in attendance.

The address of welcome was given by Dr. L. Gilman, one of the oldest dentists in the State. The papers were all interesting and were fully discussed.

Thursday afternoon (the 16th) was devoted to clinics. Dr. J. Lenox Curtis, of New York, removed an incisted lower wisdom tooth, which, when reached, was found with the crown turned down, bottom side up.

Dr. Lovejoy, of Montreal, showed a beautiful piece of removable bridge work in the mouth of a patient, it being the lower second bicuspid and first molar, fitting over gold caps on the first bicuspid and second molar.

Dr. Dwight M. Clapp, of Boston, demonstrated his method of combination filling as described in his talk (published on another page). Dr. W. R. Blackstone his method of rapid gold filling using soft and cohesive gold.

Dr. J. E. Waitle, of Boston, showed the use of the Packard ether inhaler. Dr. Waitle also had a show-case containing several specimens of abnormally shaped teeth and pieces of necrosed bone, a very interesting collection.

Thursday evening was devoted to a banquet, at which about ninety, including several ladies, sat down. After the edibles had been served and discussed came the post-prandial exercises. Dr. George F. Cheney, president of the society, acted as toastmaster, and first called on the veteran, Dr. James Lewis, of Burlington, for "a story." The call was responded to in a very entertaining and witty manner, which brought down the house. When the applause had subsided, Dr. A. J. Parker, of Bellows Falls, was asked to speak on "The opportunities of the Dentists," which he did in a most entertaining manner. Dr. Dwight M. Clapp, of Boston, followed with remarks on "New England Dentistry." The Sentiment, "The Dental Board of Quebec," brought its president, Dr. Stephen Globuesky, of Montreal, to his feet, and he made a very happy speech, in which he acknowledged the courtesies extended him and the other members of the profession from the Dominion, by the Vermont Society. Mr. Frederick W. Bancroft, of Montpelier, was then introduced, and sang a solo in his inimitable style. In this connection it should be said that the Executive Committee had arranged for several songs by Mr Bancroft, both solos and as a member of a male quartette, but the lateness of the hour compelled the omission of all but this one; it also caused to be dropped from the programme the violin solos arranged to be given by Dr. K. Longfellow Cleaves, of Montpelier, and it can be asserted that the assembled company missed a rare musical treat when these portions of the post prandial exercises were omited. Mr. Bancroft's effort was liberally applauded and then followed remarks: "Dental Education of the Dominion," by Dr. W. G. Beers, of Montreal. Dr. J. E. Waitle, of Boston, responded for the honorary members of the society, and Dr. J. L. Perkins, most happily answered for the ladies. This closed the literary part of the programme and the banqueters retired well satisfied that there had nothing been left undone on the part of the Executive Committee to make the occasion enjoyable.

The committee greatly regretted that lack of time prevented the reading of a poem written for the occasion by Mr. W. S. Flinn, and the company lost a good thing by not hearing it.

Among the visitors were Dr. G. Lenox Curtis, New York; Drs. W. Geo. Beers, A. H. Beers, J. A. Bazin, Geo. W. Lovejoy, J. H. Bourbon, Stephen Globueskey, J. B. Ridout, of Montreal; Dr. C. H. Wells, of Huntington, P. Q.; Dr. S. S. Stowell, Pittsfield, Mass.; Dr. C. P. Webster, Franklin Falls, N. H.; Dr. W. R. Blackstone, Manchester, N. H.; Dr. G. A. Young, Concord, N. H.; Dr. F. S. Putnam, Newport, N. H.; Dr. and Mrs. J. E. Waitle, and Dr. D. M. Clapp, of Boston, Mass.; Dr. J. H. Collins, Grandville, N. Y., and Dr. C. R. Hall, of Westport, New York.

Friday morning the following officers were elected: President, Dr. A. J. Parker; 1st Vice President, Dr. W. H. Wright; 2nd Vice-President, Dr. E. O. Blanchard; Secretary, Dr. Thomas Mound; Treasurer, Dr. W. H. Munsell; Executive Committee, Drs. G. O. Webster, C. W. Staples and F. P. Mather.

After listening to a paper by C. W. Steele, the meeting was adjourned to meet at White River Junction, the third Wednesday in March 1894.

